# Aging Mechanism and Properties of SBS Modified Bitumen under Complex Environmental Conditions

**DOI:** 10.3390/ma12071189

**Published:** 2019-04-11

**Authors:** Hui Wei, Xianping Bai, Guoping Qian, Feiyue Wang, Zhengfu Li, Jiao Jin, Yuhao Zhang

**Affiliations:** 1 State Engineering Laboratory of Highway Maintenance Technology, Changsha University of Science & Technology, Changsha 410114, Hunan, China; xianpingbai@126.com (X.B.); guopingqian@sina.com (G.Q.); jinjiao@csust.edu.cn (J.J.); 18390975052@163.com (Y.Z.); 2 School of Civil Engineering, Central South University, Changsha 410083, Hunan, China; wfyhn@163.com; 3 Department of Municipal Design, Zhonglugang Engineering Conultants, Beijing 100029, China; Lzf199405@163.com

**Keywords:** SBS modified bitumen, ultraviolet aging, physical properties, rheological properties, microstructure

## Abstract

Bitumen aging can lead to the deterioration of asphalt pavement performance, shortening the service life of road. In order to solve the problem that current studies on the ultraviolet (UV) aging of bitumen either ignore the effects of natural environmental conditions or only consider the effects of water. In this study, different aqueous media and UV coupled simulated aging tests were carried out on virgin bitumen and styrene butadiene styrene (SBS) modified bitumen in a UV environment chamber. The combination of macroscopic performance tests and microstructure tests was used to analyze the physical, rheological, and microstructure changes of virgin bitumen and SBS modified bitumen after The film oven test (TFOT) aging and UV aging in different environments (UV, UV + Water, UV + Acid, UV + Salt). Dynamic shear rheometer (DSR) results indicated that UV aging results in the increase of rutting factor and the improvement of rutting resistance at high temperature. The Fourier transform infrared spectrum (FTIR) results illustrated that the bitumen would be oxidized and SBS would be degraded under ultraviolet radiation. The four-component analysis test results showed that light component migrated to the heavy component during the aging process. Moreover, water will aggravate the UV aging of bitumen, and the presence of acid or salt worsens ultraviolet aging.

## 1. Introduction

As a kind of material with superior performance, styrene butadiene styrene (SBS) modified bitumen has been widely used in pavement construction and maintenance [1,2]. However, the performances of SBS modified bitumen deteriorate under unfavorable environmental conditions and vehicle loads, which is partly due to aging under ultraviolet (UV) radiation and high temperature environment [3,4,5]. Bitumen aging can be divided into thermal aging and photoaging [6,7]. Thermal aging mainly occurs in mixture mixing, transportation, paving, and other pavement construction stages. Photoaging, which is mainly caused by ultraviolet radiation, mainly occurs in the pavement service life. The aging properties of bitumen or bituminous mixtures are always illustrated based on the results of a UV aging test that either ignores the effects of natural environmental conditions or only considers the effects of water. The result that was obtained under such conditions failed to precisely reveal the aging properties of bitumen or bituminous mixtures. Therefore, it is essential to reveal the aging mechanism and the properties of SBS modified bitumen under complex conditions.

The pavement is directly exposed to atmosphere, vehicle load, and natural environmental factors (water, biochemical corrosion, industrial gas, bacteria, etc.) that can promote or inhibit the aging during the long-term photoaging process [8,9]. Acid rain, a kind of acidic deposition, is able to cause serious damage on the surface of building facilities, especially in the southwest of China [10,11]. In addition, salts (such as deicing salts, sea salt) are also more corrosive to the road surface. For example, more than 1000 km of arterial highway is close to or even goes across the sea in some regions of China. Accordingly, it is inevitable that seawater will invade the pavement structure under the effect of tide and storm [12]. Furthermore, salting on the road surface is a more effective and commonly technique of deicing and snowing, but this will cause the road in the seasonal frozen region to suffer from salt corrosion. However, the present research on bitumen aging mainly focuses on high temperature and light, and rarely considers the damage that is caused by water, acid, and salt in the natural environment [13,14,15]. Most of the relevant researches considering environmental factors are mostly focused on the impact of cement-based materials [16,17], but less on the impact of bitumen materials. The American Society for Testing and Materials (ASTM D4798 2004) studied the effects of weathering of heat, light, and water on the aging of bitumen materials, and proposed corresponding accelerated aging specifications for asphalt materials, but they did not conduct comprehensive research on water, acid, and salt materials [18]. Hence, it is of great importance to understand the influence of water, acid, and salt on the durability of bitumen systematically, which is also necessary to reveal the localized deterioration mechanism.

The (rolling) thin film oven test ((R)TFOT) test and the pressure aging vessel (PAV) test are commonly used in the world to simulate the short-term thermo-oxidative aging and long-term aging behavior of bitumen [13,19]. However, due to ignoring the effect of ultraviolet radiation, the correlation between PAV simulation aging and actual pavement aging is not significant. Although all kinds of unfavorable factors of natural environment species are fully considered in the natural aging test, the aging process lasts for a long time, and the experiment has big error and poor reproducibility, so it is difficult to carry out any single-factor or multi-factor control aging research. Some scholars have carried out the laboratory simulated bitumen UV aging test through the self-developed UV environment chamber, and the indexes of conventional performance, rheological property, and functional group change before and after aging were selected to analyze and evaluate the aging behavior of bitumen [20,21,22,23,24,25]. The authors found that, under different aging methods, the aging behavior of modified bitumen of different materials is very different, and the functional indicators before and after aging greatly vary. Laboratory simulated aging has the characteristics of short time, strong controllability, and good reproducibility.

The existing research hold that the aging mechanism of bitumen is mainly oxidation, physical hardening that is caused by recombination and molecular structure change, volatilization of light components, and selective absorption or adsorption of bitumen components by aggregates [6,26,27]. Among them, oxidation is the main cause of aging, whether it is heat aging or photoaging. However, for areas such as abundant rainfall regions, severe acid rain regions, frozen area demineralized with chloride salt, and saline-alkali regions rich in mineral salts, the mechanism of water, acid, and salt on the accelerated aging of bitumen is still in a blank.

Therefore, in this paper, based on the procedure of TFOT aging, UV aging tests of virgin bitumen and SBS modified bitumen were carried out under four types of environmental conditions, they were UV, UV + Water, UV + Acid, and UV + Salt. The scanning electron microscope (SEM), Fourier transform infrared spectrum (FTIR), and four-component method were adopted to analyze the aging mechanism, while the dynamic shear rheometer (DSR) and Bending beam rheometer (BBR) were employed to reveal the aging properties of SBS modified bitumen under different test conditions.

## 2. Materials and Methodology

### 2.1. Materials

The virgin bitumen that was used in our research is road petroleum bitumen with a penetration grade of 70 and a quality grade of A produced by Maoming Branch of Sinopec, and the SBS modifier is linear structure produced by Yanshan Petrochemical Company of China. Table 1 and Table 2 shows the main physical properties of virgin bitumen and SBS.

### 2.2. Preparation of SBS Modified Bitumen

The virgin bitumen in melting state (135 °C) was mixed with 5% (mass of the virgin bitumen) SBS modifier, the SBS was evenly dispersed to the virgin bitumen by hand stirring for 2–3 min, and then the blend was heated to 160 °C in a multipurpose electric furnace (CS-2.5, Xinghua Macro Industry Electric Equipment Factory, Jiangsu, China). Afterwards, it was subjected to rotary shearing at 4000 r/min for 60 min at 160 °C while using a high-speed shear (FM300, Shanghai FLUKO Technology Development Co., LTD., Shanghai, China). After the rotary shearing was completed, it was placed in an oven (101-II, Cangzhou Dongda Test Instrument Co., LTD., Hebei, China) at 160 °C for swelling for 1.5 h to obtain SBS modified bitumen.

### 2.3. Preparation of Water, Acid and Salt Solutions

**Figure 1:**
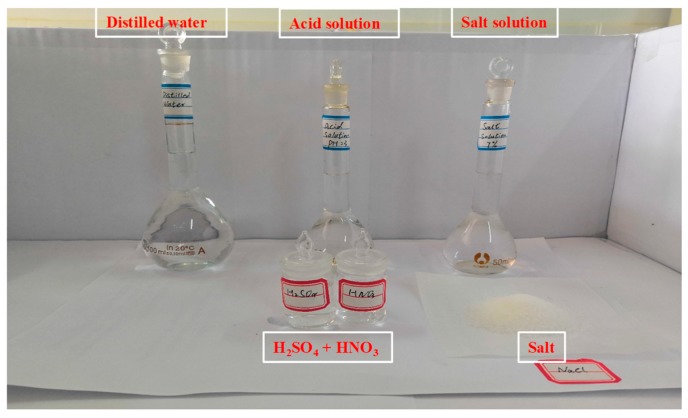
Simulated environment reagents.

A distillation machine prepared the distilled water (Shenzhen Yiliyuan Water Treatment Equipment Co., LTD., Shenzhen, China). The main components of acid rain are NH^4+^, Ca^+^, Na^+^, K^+^, Mg^+^, H^+^, SO_4_^2−^, HSO_4_^2−^, NO_3_^−^, HCO_3_^−^, Cl^−^, etc. The concentration of ${\mathrm{SO}}_{4}^{2-}$ in acid rain is 5–10 times higher than that of ${\mathrm{NO}}_{3}^{-}$. In this paper, the acid solution was prepared by analytically pure sulfuric acid and nitric acid and distilled water, according to ${\mathrm{SO}}_{4}^{2-}:{\mathrm{NO}}_{3}^{-}$ = 9:1 and PH = 3 [28,29], which is measured by PH-meter (PHS-25, Shanghai INESA Scientific Instrument Co., Ltd., Shanghai, China). Sodium chloride crystals and distilled water at a concentration of 7% prepared the salt solution. Figure 1 shows the distilled water and two solutions.

### 2.4. Aging Procedure

**Figure 2:**
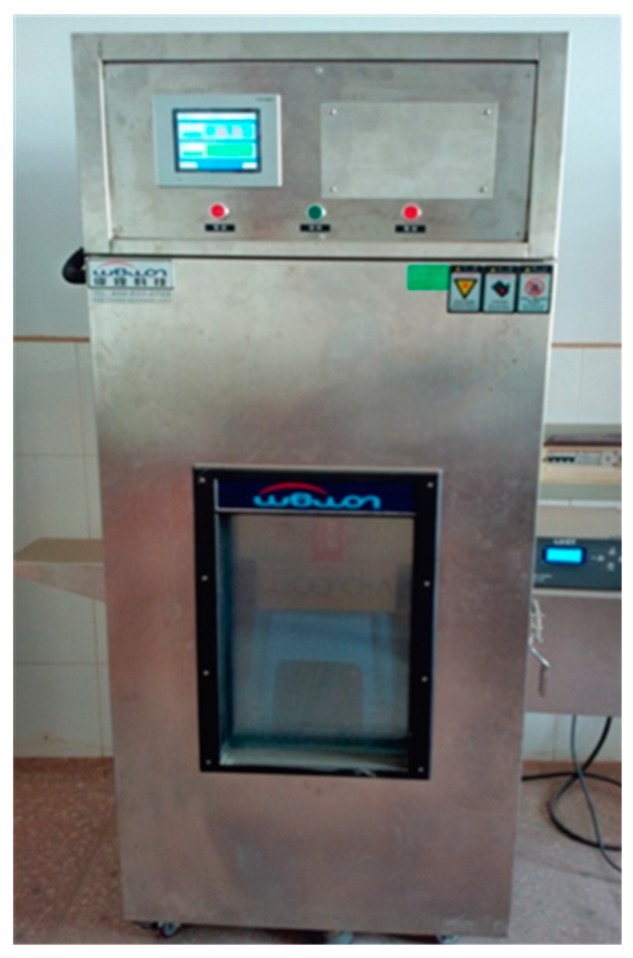
Ultraviolet (UV) environment chamber.

The film oven test (TFOT) was selected to simulate the thermal oxidation of bitumen during mixing and paving of bituminous mixtures according to ASTM D1754 (2014). To ensure that the thickness of the bitumen film was about 3 mm, 50 ± 0.5 g of virgin bitumen or SBS modified bitumen was placed on a Φ140 ± 0.5mm iron pan. Subsequently, the bitumen film specimens were placed in a film oven to rotate at a speed of 5.5 r/min and aged for five hours (163 °C). The samples of SBS modified bitumen and virgin bitumen after TFOT aging were exposed to ultraviolet for seven days in a self-made ultraviolet environment simulation chamber (see Figure 2). The environment chamber adopts a LED cold light source, the main ultraviolet t wave is 365 nm, and the temperature is 25 °C. The SBS modified bitumen and virgin bitumen UV aging test is set to four modes, namely UV, UV + water, UV + acid, and UV + salt. For the UV + water, UV + acid, light + UV mode, and spray 1 g of water/acid/salt on the surface of the bitumen sample on a daily basis to simulate the adverse environmental conditions of the asphalt pavement. 

### 2.5. Physical Properties Test

At present, the penetration, softening point, and viscosity are used as the main indexes to evaluate bitumen performance in most countries. The physical characteristics of the SBS modified bitumen and virgin bitumen were detected, for instance, the penetration (25 °C) and softening point, according to ASTM D36, ASTM D5, respectively.

### 2.6. Dynamic Shear Rheometer (DSR)

A dynamic shear rheometer (Physica MCR 301, Anton Paar Instruments, Ostfildern, Germany) was applied to conduct temperature sweep tests on virgin bitumen and SBS modified bitumen under different aging state according to ASTM D7175. All of the tests were performed using constant-strain mode at a fixed frequency of 10 rad/s. In order to ensure the deformation of the bitumen sample within the nonlinear viscoelasticity range, as for the virgin bitumen, the strain control values of the temperature sweep test for the original sample, the TFOT sample, and the UV-aged sample were 12%, 10%, and 1%, respectively. Correspondingly, the ones for SBS modified asphalt were 3%, 3%, and 1%. The temperature ranged from 40 °C to 90 °C with an increment of 2 °C /min. The gap and the diameter of parallel plates were 1 mm, and 25 mm, respectively. Rheological parameters, such as complex shear modulus (|G^*^|), phase angle (*δ*), and rutting factor (|G^*^|/sin *δ*) as a function of temperature for all samples were applied to evaluate the rheological properties of the bitumen in the aging process.

### 2.7. Fourier Transform Infrared Spectroscopy (FTIR)

Fourier transform infrared spectroscopy (FTIR) is considered as one of the most promising employed technique to detect the chemical functional groups that are present in polymer chains. In general, different functional groups correspond to absorption peaks at different wavenumbers in the infrared spectrum, and the materials can be qualitatively and quantitatively analysed according to the appearance of the absorption peaks and their intensity and peak area. In this paper, the Bruker Tensor 27 Fourier transform infrared spectrometer (Bruker Corporation, Karlsruhe, Germany) was used to characterize the functional group changes of polymer modified bitumen after aging. The spectra were obtained, ranging from 4000 cm^−1^ to 500 cm^−1^ with a resolution of 1~0.4 cm^−1^. The wavenumber accuracy is 0.01/2000 and the absorption precision is 0.01%.

### 2.8. Scanning Electron Microscope (SEM)

As an important research method in material science, scanning electron microscope (SEM, S-3000N, Hitachi Limited, Tokyo, Japan) is used to observe the morphology and structure of materials. The principle is that, by emitting high-energy electron beam to the sample, the electron beam will produce the secondary electron and the scattered electron, and so on, and the information will be changed from the optical signal to the electric signal through the systematic processing. After the video amplifier is amplified, the image of the sample surface can be formed on the screen of the picture tube. In this paper, in order to observe the surface cracking characteristics of the aged bitumen samples, scanning electron microscope (S-3000N, HITACHI, Japan) was operated at 5 kV, with a magnification of 300. The specimens were successively sputter coated with a thin gold film prior to making specimens charged for SEM observation.

## 3. Results and Discussion

### 3.1. Physical Property

Table 3 summarizes the effects of UV radiation on the physical properties of SBS modified bitumen and virgin bitumen in different environments. UV aging results in the decrease of penetration and the increase of the softening point of virgin bitumen and SBS modified bitumen. It is due to the migration of light components of bitumen to heavy components by ultraviolet radiation, the breaking of the C=C double bond of the elastic polybutadiene chain in SBS, and the degradation of SBS. As a result, SBS modified bitumen becomes harder and brittle after aging, so its penetration decreases and softening increases. The effect of acid or salt medium on the softening point and penetration of SBS modified bitumen is greater than that of pure water medium, and the change of penetration and softening point of the two kinds of bitumen is the least under ultraviolet radiation in the dry environment. Aging will lead to the bitumen to become hard and brittle, so the penetration of the virgin bitumen and SBS modified bitumen decreases and the softening point increases. It is indicating that moisture will accelerate the UV aging of bitumen, acid, or salt will further accelerate the corrosion and aging of bitumen, and further affects its rheology and mechanics. It is also believed that water, acid, and salt can invade into pavement through the cracks and cause more serious damages to the pavement structure as the micro-cracks appear [12].

### 3.2. Rheological Properties

**Figure 3:**
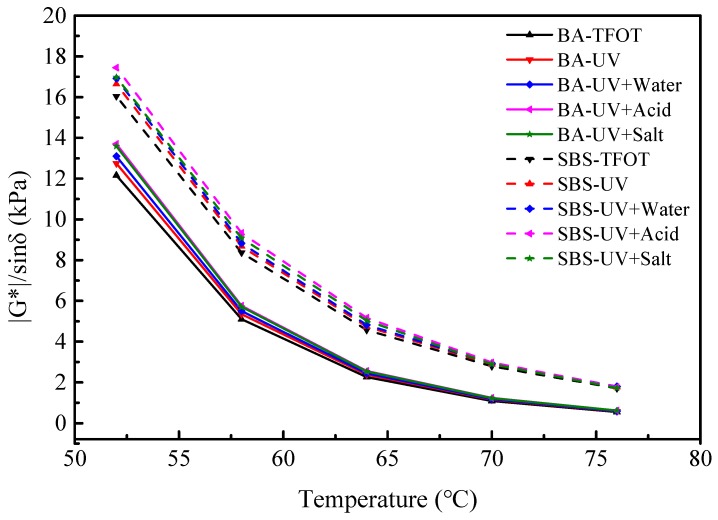
Rutting factors of bitumen before and after UV aging in different environments.

The high temperature rheological properties of virgin bitumen and SBS modified bitumen after short term aging, UV aging, and UV aging of different moisture media are shown in Figure 3 (BA and SBS represent virgin bitumen and SBS modified bitumen, respectively). Strategic highway research program recommends using the rutting factor (|G^*^|/sin *δ*) to evaluate the high temperature rutting resistance of bitumen. The greater the |G^*^|/sin *δ* is, the better the high temperature rutting resistance performance. The rutting factor of virgin bitumen and SBS modified bitumen has been improved after UV aging and UV aging of different moisture medium, and the one of acid medium is the highest. UV aging increases the rutting factor (|G^*^|/sin *δ*) of bitumen, which has a certain improvement effect on the high temperature performance of bitumen.

In sum, the effect of water, acid, and salt cannot be negligible for the bitumen during the aging process. In acidic media, on the one hand, acidic substances, such as carboxylic acids and phenols in the bitumen and aging products of bitumen are dissolved and ionized, and on the other hand, H^+^, ${\mathrm{SO}}_{4}^{2-}$, and ${\mathrm{NO}}_{3}^{-}$ in the acid solution react with the active groups in the bitumen, so that the molecular bonds of bitumen are broken and destroyed. The dual action of dissolved radiation and chemical corrosion accelerates the aging of the bitumen. In salt media, the salt will accumulate in the surface of bitumen during the evaporation of water, and change the continuous state of materials [12]. Although bitumen has been regarded as a kind of waterproof material, moisture will also enter the interior of the bitumen with the generation of micro-cracks on the bitumen surface. Under UV radiation, the saturated and aromatic components of bitumen are slightly dissolved in water, so the light component of the bitumen decreases, which accelerates the aging of the bitumen. In conclusion, water, acid, and salt can accelerate the ultraviolet aging of bitumen.

### 3.3. Four-Component Analysis

**Figure 4:**
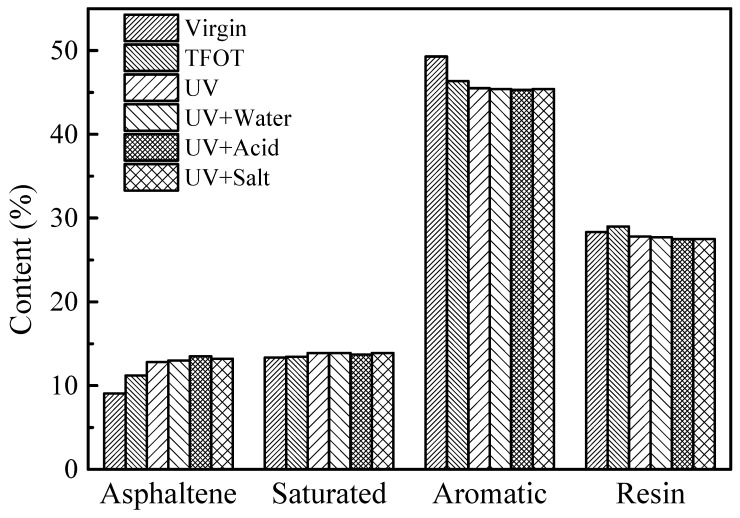
Contents of four components of virgin bitumen before and after UV aging in different environments.

**Figure 5:**
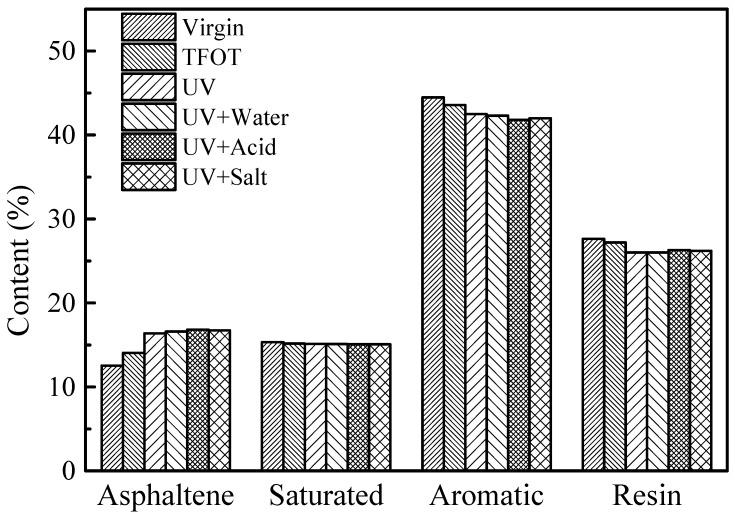
Contents of four components of SBS modified bitumen before and after UV aging in different environments.

The contents of four components of virgin bitumen and SBS modified bitumen after short-term aging, UV aging, and UV aging of different moisture media are shown in Figure 4 and Figure 5. After UV aging, the changes of the four components of SBS modified bitumen and virgin bitumen are basically the same. Ultraviolet radiation results in the change of four components’ content of bitumen, with the saturated content basically remaining the same, resin and aromatic content decreasing slightly, and the asphaltene content increased. Changes in the four components content of the bitumen will inevitably result in changes in the macroscopic properties of the bitumen. The results of the four-component analysis test are consistent with the results of the DSR test and the physical property test. Asphaltene is the heavy component with the largest molecular weight among the four components. During the aging process, some low molecular weight substances will undergo polycondensation and migration to heavy components, so the asphaltene content changes the most. The relative density of the saturated fraction is the lowest. In the process of bitumen aging, the chain breaking reaction mainly occurs in saturated fractions, forming small molecules with a low boiling point and being easy to volatilize. However, due to the relatively small content of the saturated fraction, the amount of volatilization is not obvious, so the content of saturated fraction is basically the same. In the aging process, the aromatics were converted into resin and the resin was transferred to asphaltene, so the aromatic content decreased while the asphaltenes content increased. As an intermediate product, the content of resin was affected by the rate of formation and consumption, and it then decreased slightly. For asphaltene, the content of ultraviolet radiation in aqueous medium is higher than that in pure ultraviolet radiation, especially in acid medium. For aromatic fraction, in the four ultraviolet radiation modes, the aromatic content in the acidic medium mode is the smallest, which indicates that the acidic solution has a more pronounced effect on the aromatic aging of the bitumen. It is due to the dissolution and ionization of the acidic species in the bitumen and the chemical reaction with the reactive groups in the bitumen and H^+^, ${\mathrm{SO}}_{4}^{2-}$, and ${\mathrm{NO}}_{3}^{-}$ in the acid solution.

### 3.4. Functional Group Characteristics

**Figure 6:**
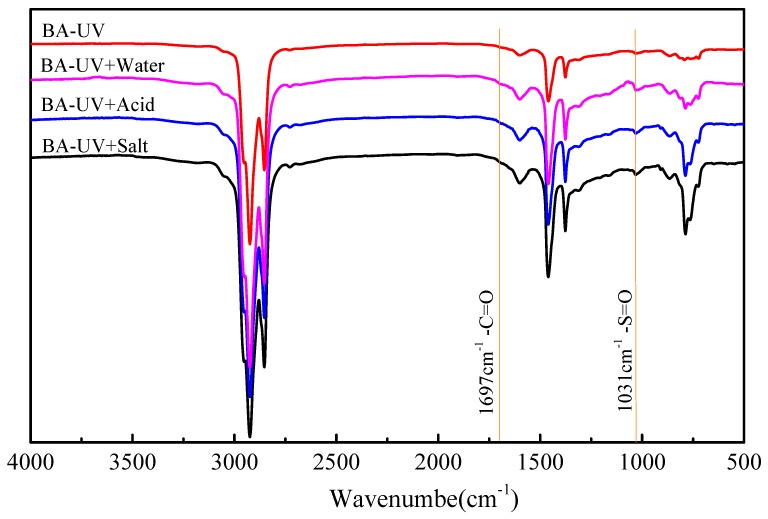
Fourier transform infrared spectroscopy (FTIR) spectra of base bitumen before and after UV aging in different environments.

**Figure 7:**
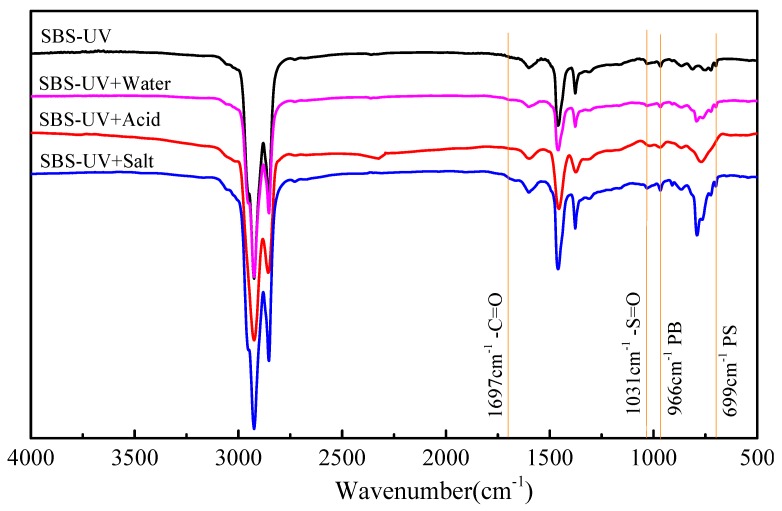
FTIR spectra of SBS modified bitumen before and after UV aging in different environments.

The infrared spectra of virgin bitumen and SBS modified bitumen after short term aging, UV aging, and UV aging of different moisture media are shown in Figure 6 and Figure 7. Carbonyl (C=O) and sulfoxide (S=O) can be used as a symbol of bitumen aging [24,30]. Under ultraviolet radiation, the single bond C-O in bitumen absorbs ultraviolet and become from ground state to excited state and produces active free radicals. These free radicals are so active that they easily react with oxygen to form C=O. It is generally believed that the carbonyl group content increases and the carbonyl absorption peak intensity increases after UV aging. Figure 6 and Figure 7 show that the characteristic peak of carbonyl group is at 1697 cm^−1^. However, the carbonyl characteristic peaks of the virgin bitumen and SBS modified bitumen are not obvious after ultraviolet radiation, which may be due to the fact that the ultraviolet radiation time is too short to cause the severe photoaging of bitumen. Generally, the carbonyl index and sulfoxide index can be used to evaluate the aging behavior of bitumen. However, the change of the characteristic functional group absorption peak in this test is relatively small. If the test error is not taken into account, then there also will be a big error in the calculation of the absorption peak of carbonyl and sulfoxide groups. Therefore, the characteristic functional group index is not used to evaluate the aging behavior of bitumen in this paper. The characteristic sulfoxide functional group appears at 1030 cm^−1^. The sulfoxide absorption peak of virgin bitumen is slightly stronger than that of the SBS modified bitumen, which indicates that the aging of virgin bitumen is more serious. The intensity of the sulfoxide-based absorption peaks of bitumen in different environments change lightly, and the intensity ranks as UV < UV + Water < UV + Acid or UV + Salt. 966 cm^−1^ and 699 cm^−1^ are the bending vibration absorption peaks of C=C double bond in polybutadiene segment (PB) and C-H in polystyrene segment (PS) benzene ring, respectively, under ultraviolet radiation. The intensity of the two-absorption peak is slightly decreased, especially under the condition of UV + Acid. This indicates that the C=C double bond of the PB segment and the C-H bond of benzene ring of PS segment are broken under ultraviolet radiation, resulting in the destruction of the original network crosslinking structure of SBS in modified bitumen, and which is the main reason for the degradation of SBS.

### 3.5. Apparent Morphology

**Figure 8:**
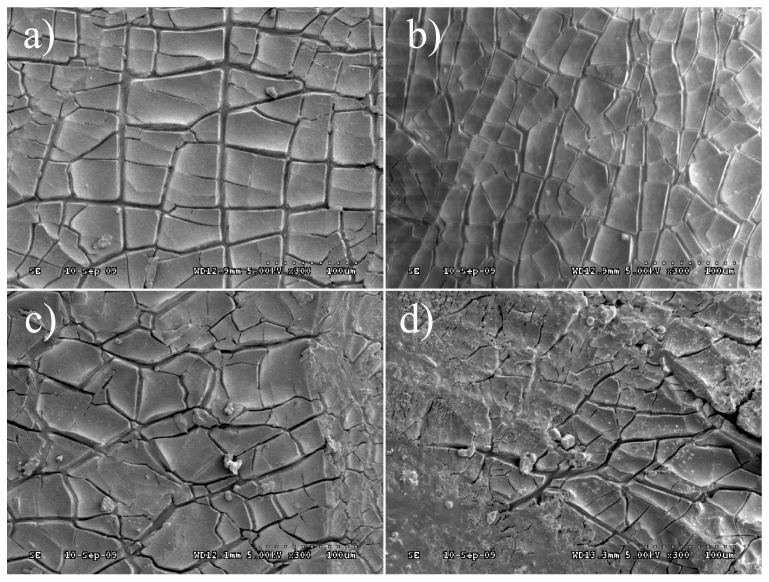
Apparent morphology of virgin bitumen after UV aging in different environments: (**a**) UV; (**b**) UV + Water; (**c**) UV + Acid; and, (**d**) UV + Salt.

**Figure 9:**
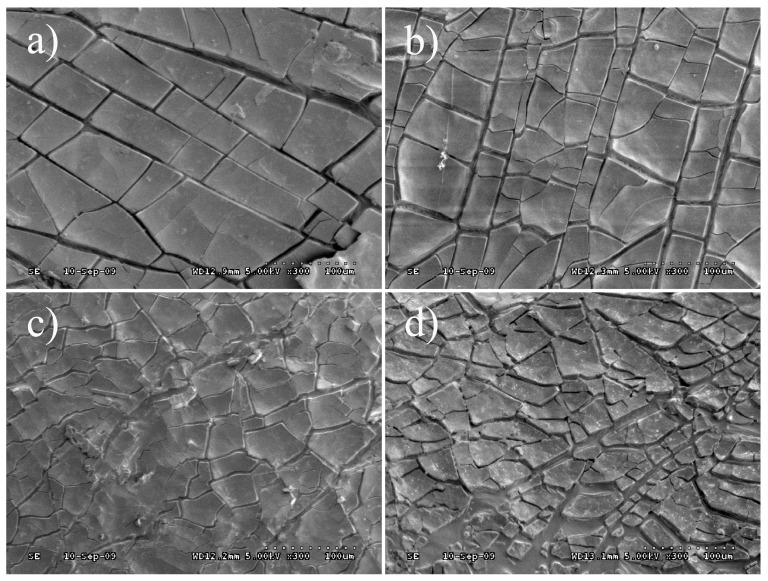
Apparent morphology of SBS modified bitumen after UV aging in different environments: (**a**) UV; (**b**) UV + Water; (**c**) UV + Acid; and, (**d**) UV + Salt.

SEM analysed the structural characteristics of virgin bitumen and SBS modified bitumen after short term aging, UV aging, and UV aging in different aqueous media. The experimental results were shown in Figure 8 and Figure 9 (magnification 300 times). Ultraviolet radiation can arouse cracking on bitumen surface, and the SBS modified bitumen crack is tidier than that of virgin bitumen under the same conditions. The cracking of the bitumen surface can be used to characterize the degree of aging. The more obvious the fragmentation of bitumen surface cracking, the more serious the aging degree. Under ultraviolet aging of the moisture medium, the cracking of the bitumen surface is further aggravated, and the surface of the virgin bitumen is more severely fragmented. The bitumen is subjected to acid corrosion in the acidic medium, the crack width of the virgin bitumen is increased, and the corners of the fragments are warped. The surface cracking of SBS modified bitumen is increased and irregular, but the crack is small. In the salt medium, the surface of the virgin bitumen is covered with salt and the crack is deep and irregular after UV aging. Generally speaking, the existence of dispersed SBS phase improves the engineering properties at the high and low temperature of bitumen. However, the surface cracking imply that virgin bitumen and SBS modified bitumen gradually become brittle at a lower temperature after UV aging [31]. In a word, the aging resistance of SBS modified bitumen is better than that of virgin bitumen in terms of surface cracking due to UV aging. Under the four environments, the bitumen aging cracking ranks as UV < UV + Water < UV + Acid or UV + Salt, which was consistent with the results of the microstructure and macro performance of bitumen after UV aging.

## 4. Summary and Conclusions

In this paper, macroscopic performance, microstructure, and apparent morphology test method were used to investigate the UV aging of virgin bitumen and SBS modified bitumen. Based on the test result and statistical analysis of bitumen at different aging states, the following conclusions can be drawn:

(1) The aging of bitumen pavement during service life is not only affected by high temperature and ultraviolet radiation. The influence of environmental factors, such as aqueous solution, on aging should not be ignored. When subjected to water, acid rain, or chlorine salt corrosion, the asphalt pavement has a higher level of aging, especially in acidic or salt environments.

(2) The aging laws of virgin bitumen and SBS modified bitumen by ultraviolet radiation are basically the same. The anti-photoaging performance of SBS is better than that of virgin bitumen.

(3) The FTIR test showed that under UV radiation, the absorption peaks of carbonyl(C=O) and sulfoxide groups(S=O) of bitumen-based increased, and the absorption peaks of PB and PS segments of SBS decreased. It indicates that the nature of SBS modified bitumen aging is the change in the molecular structure of the bitumen-based and the degradation of SBS.

(4) After UV aging, macroscopically, the bitumen showed a decrease in penetration and an increase in softening point and rutting factor. Microscopically, the light components of bitumen migrate to heavy components, resulting in a decrease in aromatic and gelatinous content, an increase in asphaltene content, and cracking on the bitumen surface.

## Figures and Tables

**Table 1:** Physical properties of 70# virgin bitumen.

Properties	Limitation	Values	Methods
Penetration (25 °C, 100 g, 5 g) (0.1 mm)	60~80	61.8	ASTM D5
Penetration index	−1.5~+1.0	0.6	ASTM D5
Softening point (°C)	≥46	47.8	ASTM D36
Ductility (15 °C, 5 cm/min) (cm)	≥100	>100	ASTM D113
Density (15 °C) (g/cm^3^)	—	1.034	ASTM D70
Dynamic viscosity (60 °C) (Pa·s)	180~240	215	ASTM D2171

**Table 2:** Physical properties of SBS copolymer.

Modifier	S/B Ratio	Oil Filling Rate (%)	Volatile Component (≤%)	Tensile Strength (≥MPa)	Elongation at Break (≥%)	Tensile Set at Break (≤%)
SBS	30/70	0	0.7	15	700	40

**Table 3:** Physical properties of virgin bitumen and SBS modified bitumen before and after UV aging in different aqueous media.

Test Index	Bitumen Type	Aging Type					
		Virgin	TFOT	UV	UV + Water	UV + Acid	UV + Salt
Penetration (25 °C, 0.1 mm)	Virgin bitumen	61.8	43.9	36.5	33.4	31.7	30.5
	SBS modified bitumen	65.0	56.0	47.6	43.0	42.3	42.5
Softening point (°C)	Virgin bitumen	47.8	53.0	54.9	55.5	57.9	56.8
	SBS modified bitumen	69.1	71.5	73.0	74.2	76.2	75.6
